# Comparative Study of the Mode of Action of Clinically Approved Platinum-Based Chemotherapeutics

**DOI:** 10.3390/ijms21186928

**Published:** 2020-09-21

**Authors:** Sarah Schoch, Sabine Gajewski, Jana Rothfuß, Andrea Hartwig, Beate Köberle

**Affiliations:** 1 Department of Food Chemistry and Toxicology, Karlsruhe Institute of Technology, Adenauerring 20a, 76131 Karlsruhe, Germany; sarah.schoch@med.lu.se (S.S.); sabine.gajewski@kit.edu (S.G.); jana.rothfuss@gmx.de (J.R.); andrea-hartwig@kit.edu (A.H.); 2 Department of Laboratory Medicine, Lund University, Scheelevägen 2, 22381 Lund, Sweden

**Keywords:** cisplatin analogues, tumor cells, gene expression profiling, DNA damage response

## Abstract

Platinum drugs are among the most effective anticancer agents, but their mode of action is still not fully understood. We therefore carried out a systematic investigation on the cellular activities of cisplatin, carboplatin and oxaliplatin in A498 kidney cancer cells. Cytotoxicity was higher for cisplatin and oxaliplatin compared to carboplatin, with induction of apoptosis as the preferred mode of cell death. Gene expression profiling displayed modulation of genes related to DNA damage response/repair, cell cycle regulation and apoptosis which was more pronounced upon oxaliplatin treatment. Furthermore, repression of specific DNA repair genes was restricted to oxaliplatin. Transcriptional level observations were further analyzed on the functional level. Uptake studies revealed low intracellular platinum accumulation and DNA platination upon carboplatin treatment. Removal of overall DNA platination was comparable for the three drugs. However, no processing of oxaliplatin-induced interstrand crosslinks was observed. Cisplatin and carboplatin influenced cell cycle distribution comparably, while oxaliplatin had no effect. Altogether, we found a similar mode of action for cisplatin and carboplatin, while the activity of oxaliplatin appeared to differ. This might be clinically relevant as due to the difference in mode of action oxaliplatin could be active in tumors which show resistance towards cisplatin and carboplatin.

## 1. Introduction

**Figure 1:**
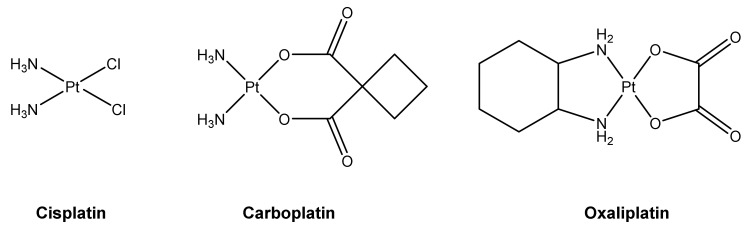
Structures of the clinically approved platinum-based anticancer drugs cisplatin, carboplatin and oxaliplatin.

Cisplatin is one of the most active chemotherapeutic drugs used for the treatment of various solid neoplasms, including testicular, bladder, lung and head and neck cancer [1]. However, its clinical utility is restricted by severe side effects, especially acquired or intrinsic tumor cell resistance and dose-limiting nephrotoxicity [2]. To overcome these limitations, great efforts have been made to search for cisplatin analogues which are better tolerated by patients and/or show anticancer activity in cisplatin resistant tumors. Modification of the structure of cisplatin led to the development of carboplatin and oxaliplatin (Figure 1) [3]. In various cancer cell lines, carboplatin shows less toxicity than cisplatin at equimolar concentrations [4]. It is used as first-line treatment of patients with advanced ovarian cancer, and is also clinically useful for the treatment of a number of other types of cancer, such as advanced small cell and nonsmall cell lung cancer, while it shows inferior activity than cisplatin in germ cell tumors and bladder and head and neck cancer [5]. Oxaliplatin has a different pattern of activity to that of cisplatin, and proved to be active in some tumor cells which are resistant to cisplatin [6]. It is the latest platinating drug to have been granted worldwide approval, and is used in combination with 5-fluorouracil for the treatment of metastatic colorectal cancer, which is insensitive to treatment with cisplatin and carboplatin.

Even though cisplatin, carboplatin and oxaliplatin play a major role in tumor therapy, there is a huge interest in the development of new, more efficient platinum-based antitumor agents which might broaden the range of treatable tumors and/or be better tolerated by patients [7,8]. To further develop the field of platinum-based therapy, it is, however, essential to better understand the mode of action of the established platinum therapeutics, as an understanding of how these therapeutics induce their antitumor effects would help in the development of new drugs that show activity in tumors which remain unresponsive to present platinum drugs and exhibit fewer unwanted side effects. Due to their respective structural characteristics, established platinum-based compounds are supposed to cause a differing pattern of responses in cells and also in the whole body, but the exact mechanism of their antitumor activity is still not known. Especially for oxaliplatin, knowledge about the mode of action is still scarce. There is evidence that platinum compounds mediate their antitumor activity by damaging DNA [9]. Furthermore, protein platination, which might play a role in the pharmacological characteristics of platinum compounds, has been reported [10,11]. In the present study, we carried out a thorough investigation of the mechanisms of action of carboplatin and oxaliplatin in the renal cell carcinoma cell line A498. The origin of A498 cells is the proximal tubule of a clear cell renal cell carcinoma, and these cells are widely used in cancer research since they belong to the NCI-60 panel [12]. The proximale tubule is also the site of cisplatin-induced damage and inflammation, which is the underlying cause of dose-limiting severe nephrotoxicity observed for this drug. The mode of action of cisplatin was investigated using A498 cells and published previously [13]; data relevant to the current investigation are included in the present study for comparison. The cellular activity of platinum drugs was analyzed on the transcriptional level using gene expression analysis, and observations on the transcriptional level were further examined at the functional level for confirmation. Our results show that cisplatin and carboplatin share a comparable mode of action, while oxaliplatin has a distinct pattern of activity. 

## 2. Results

### 2.1. Cytotoxic Potential of Platinum-Based Compounds 

**Figure 2:**
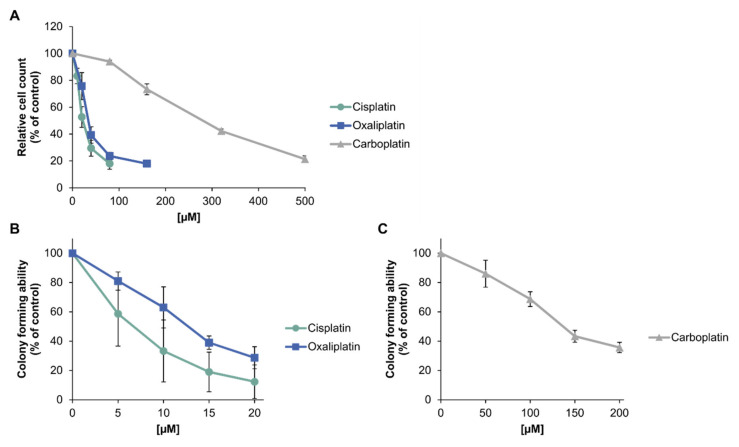
Cytotoxicity of platinum compounds in A498 cells. (**A**): Relative cell count (RCC) after treatment with cisplatin, carboplatin or oxaliplatin. A498 cells were incubated for 1 h with cisplatin (10, 20, 40, 80 µM), carboplatin (80, 160, 320, 500 µM) or oxaliplatin (20, 40, 80, 160 µM). After 72 h postcultivation, cell count was determined. RCC was obtained by normalization to the untreated control. The mean values are shown of three independent experiments ± standard deviation. (**B**)/(**C**): Colony formation ability (CFA) of A498 cells after treatment with (**B**) cisplatin or oxaliplatin or (**C**) carboplatin. Cells were incubated for 1 h with cisplatin (5, 10, 15, 20 µM), oxaliplatin (5, 10, 15, 20 µM) or carboplatin (50, 100, 150, 200 µM). Afterwards, cells were cultivated for 12 days for colony formation. Colonies were counted and the treated samples were normalized to the untreated control. The mean values are shown of three independent experiments ± standard deviation.

To investigate the toxic potential of platinum-based compounds in tumor cells, A498 kidney cancer cells were treated with the respective drug for 1 h. Acute toxicity was determined by relative cell count (RCC) and long-term toxic effects were measured by colony formation ability (CFA) (Figure 2). Regarding relative cell count (Figure 2A), cisplatin and oxaliplatin showed a similar cytotoxic potential in A498 cells, represented by an IC_50_ of 27 µM for cisplatin and 36 µM for oxaliplatin (Table 1). Carboplatin, on the other hand, showed a comparable effect only at concentrations tenfold higher than cisplatin (IC_50_ = 273 µM). Regarding the long-term cytotoxic effects, oxaliplatin was slightly less toxic than cisplatin, with an IC_50_ of 12 µM compared to 6 µM for cisplatin (Figure 2B, Table 1). However, the standard deviations of both compounds need to be taken into account, since they overlap. To achieve a similar long-term toxic effect, carboplatin needed to be applied in over tenfold higher concentrations than cisplatin (Figure 2C, Table 1).

### 2.2. Intracellular Accumulation and DNA Platination

To investigate intracellular platinum accumulation, A498 cells were treated for 2 h with 50 µM of the respective compound, and the intracellular platinum amount was measured by AAS (Table 2). Cisplatin treatment resulted in an intracellular accumulation of 23 ng Pt/10^6^ cells, while oxaliplatin treatment led to 14.9 ng Pt/10^6^ cells. For carboplatin, 4.8 ng Pt/10^6^ cells were observed when cells were treated with 50 µM of the drug, while incubation with 300 µM carboplatin showed with 25 ng Pt/10^6^ cells a similar intracellular platinum level as cisplatin. As DNA is the primary target of activity for platinum-based compounds, DNA platination was measured by ICP-MS in A498 cells treated with 50 µM of the respective compound. The amount of platinum bound to DNA directly after a 2 h exposure is shown in Table 2. DNA platinum binding was associated with intracellular platinum levels, with high amounts of overall DNA platination measured for cisplatin (383.5 nmol Pt/g DNA) and oxaliplatin (149.5 nmol Pt/g DNA), while the amount of DNA platination following treatment with carboplatin was low, i.e., 17 nmol Pt/g DNA.

### 2.3. Gene Expression Analysis

**Figure 3:**
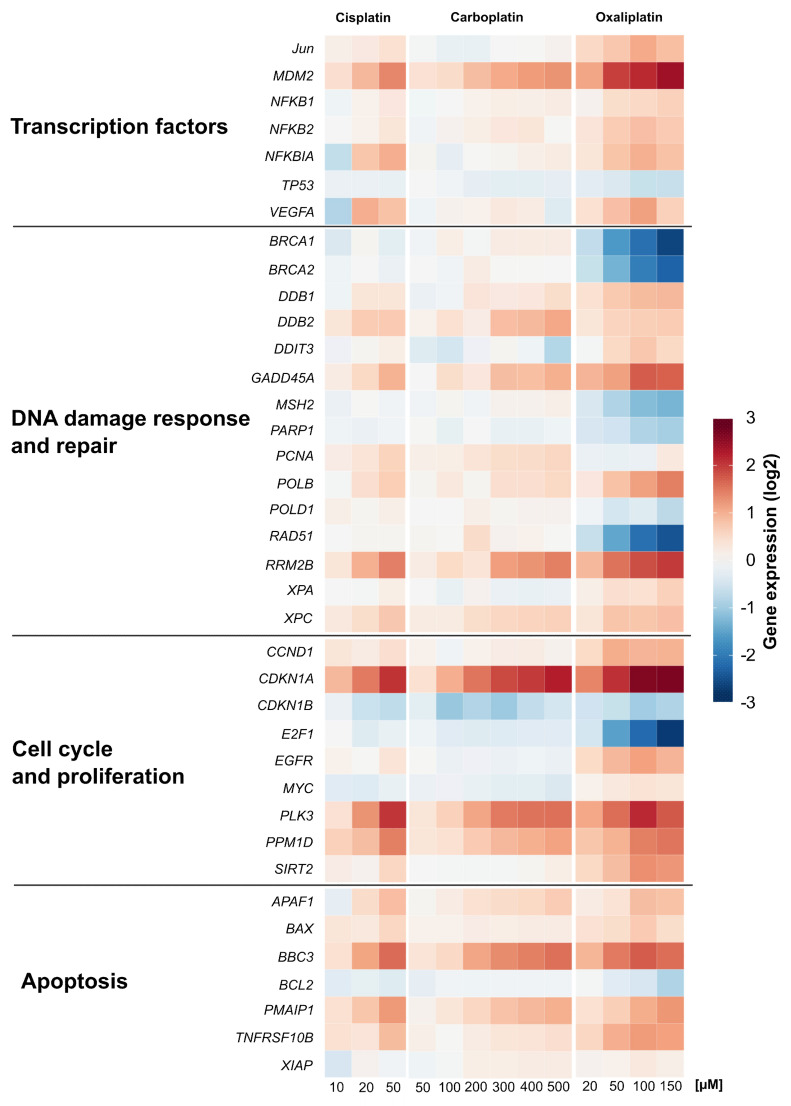
Gene expression profiling of A498 cells after treatment with cisplatin, carboplatin or oxaliplatin. Cells were treated for 1 h with various concentrations of cisplatin (10, 20, 50 µM), carboplatin (50, 100, 200, 300, 400, 500 µM) or oxaliplatin (20, 50, 100, 150 µM) followed by a postcultivation of 24 h. Gene expression was determined by high-throughput RT-qPCR. Genes were grouped into the clusters transcription factors, DNA damage response and repair, cell cycle and proliferation and apoptosis. The log2 mean values are shown for three independent experiments normalized to the untreated control whereby the control equals 0. The data of cisplatin-induced gene expression are taken from [8].

The impact of carboplatin and oxaliplatin on the gene expression profiles of genes related to genomic stability was investigated in A498 cells using high-throughput RT-qPCR [14]. The results are summarized in a heatmap in Figure 3 and compared to gene expression profiles already established for cisplatin [13]. Relative gene expressions were calculated by normalizing the treated samples to the untreated control, and are expressed as log2 values. A change of ±1 in gene expression compared to the untreated control was considered a relevant effect, but concentration-dependent trends were also considered. Carboplatin led to a gene expression pattern comparable to that of cisplatin. Oxaliplatin also increased the transcript levels of genes modulated by cisplatin. The three platinum compounds caused a concentration-dependent induction of the transcription factor MDM2 gene, the DNA damage response genes GADD45A and RRM2B, the nucleotide excision repair genes DDB2 and XPC, the cell proliferation-associated genes CDKN1A, PLK3 and PPM1D and the apoptosis-associated genes APAF1, BBC3, PMAIP1 and TNFRSF10B, indicating activation of the respective pathways. The impact on these cellular control systems was most pronounced after treatment with oxaliplatin, while cisplatin and carboplatin enhanced the transcript levels of the described genes to a similar extent. With regard to specific DNA repair genes, however, a different impact on expression was observed upon treatment with the respective platinum drug. While homologous recombination-associated genes BRCA1, BRCA2 and RAD51, the mismatch repair gene MSH2 and PARP1 which is involved in single and double strand break repair, were repressed by oxaliplatin in a concentration-dependent manner, almost no modulation of these genes was observed for cisplatin and carboplatin. Oxaliplatin also drastically decreased the expression of E2F1 gene coding for the transcription factor E2F1 which regulates the expression of genes involved in numerous cellular functions, including DNA repair.

**Figure 4:**
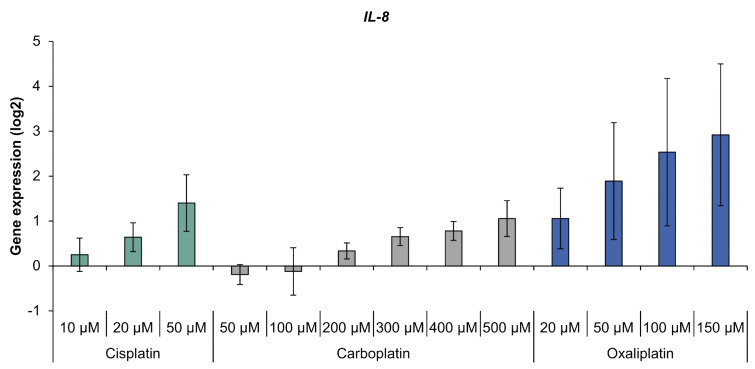
Gene expression of *IL-8* in A498 cells after incubation with cisplatin, carboplatin or oxaliplatin. Cells were treated for 1 h with cisplatin (10, 20, 50 µM), carboplatin (50, 100, 200, 300, 400, 500 µM) or oxaliplatin (20, 50, 100, 150 µM), followed by a postcultivation of 24 h. Afterwards, gene expression was analyzed using high-throughput RT qPCR. The log2 mean values are shown of three independent experiments normalized to the untreated control whereby the control equals 0 ± standard deviation. The data for cisplatin are taken from [8].

With regard to oxidative stress response, the platinum compounds had little impact on gene expression (Figure S1). Solely GPX2 coding for glutathione peroxidase was induced by cisplatin. When analyzing the inflammation marker gene IL-8, a concentration-dependent increase was observed, with the highest levels of IL-8 transcript seen with oxaliplatin treatment (Figure 4). Compared to oxaliplatin, cisplatin and carboplatin showed a moderate impact on IL-8 expression which reached a relevant level with the highest drug concentration used.

### 2.4. Impact of Platinum Drugs on p53 Levels 

**Figure 5:**
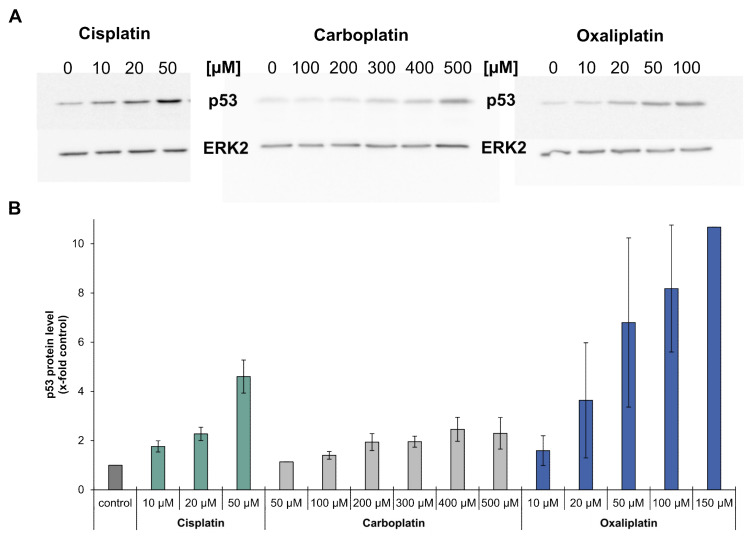
Analysis of the protein level of p53 in A498 cells after incubation with cisplatin, carboplatin or oxaliplatin. Cells were treated for 1 h with cisplatin (10, 20, 50 µM), carboplatin (50,100, 200, 300, 400, 500 µM) or oxaliplatin (10, 20, 50, 100, 150 µM). After a 24 h postcultivation period, the protein level of p53 was determined by immunoblot. (**A**): Examples of a representative immunoblot of each compound. ERK2 was used as a loading control (**B**): Semiquantification of the immunoblots. Including the loading control, the treated samples were normalized to the untreated control. With the exception of treatment with 50 µM carboplatin or 150 µM oxaliplatin, all concentrations were analyzed in three independent experiments; the results are shown as mean values ± standard deviation. N.B. 50 µM carboplatin or 150 µM oxaliplatin were analyzed only once, as no changes were seen after 50 µM carboplatin incubation or extremely high levels of p53 regarding the incubation with oxaliplatin. Results of cisplatin-induced p53 levels are taken from [8].

Gene expression analysis revealed that the platinum drugs led to an increased expression of MDM2, XPC, DDB2, GADD45A, RRM2B, CDKN1A, PLK3, PPM1D, APAF1, BBC3, PMAIP1 and TNFRSF10B. These genes are all target genes of the tumor suppressor p53 [15]. As an increased expression of p53 target genes is an indication of the activation of p53, p53 protein levels in cells treated with carboplatin or oxaliplatin were analyzed by immunoblotting and compared to findings previously described for cisplatin [13]. Incubation with carboplatin or oxaliplatin led to a concentration-dependent increase in p53 protein levels which was most pronounced for oxaliplatin, while carboplatin increased p53 levels comparably to cisplatin (Figure 5A,B).

### 2.5. DNA Damage Response and DNA Repair

**Figure 6:**
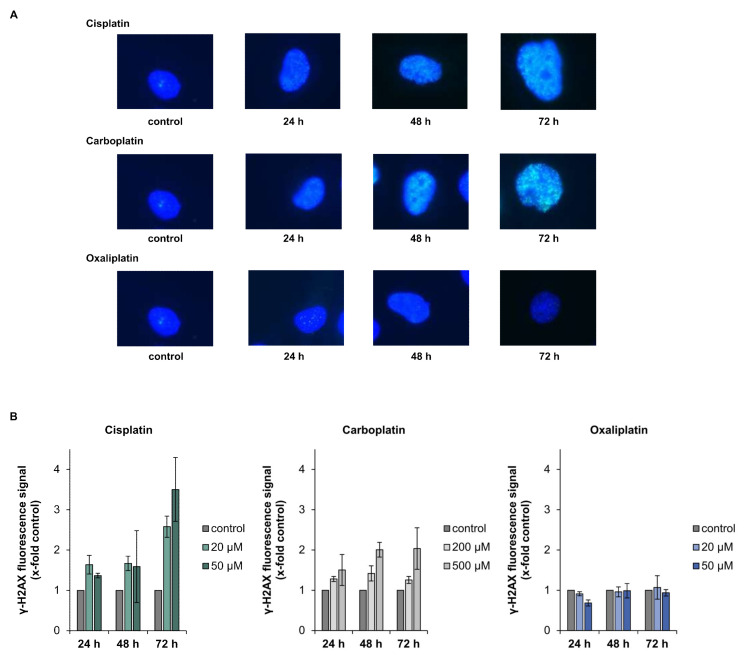
Analysis of γ-H2AX after incubation of A498 cells with cisplatin, carboplatin or oxaliplatin. Cells were treated with cisplatin or oxaliplatin (20, 50 µM) or carboplatin (200, 500 µM), followed by postcultivation of 24, 48 or 72 h. γ H2AX fluorescence was measured using fluorescence microscopy. (**A**): Representative microscope pictures of A498 cells treated with the platinum-based compounds at the highest concentration. (**B**): Quantification of the fluorescence signal. Overall γ-H2AX fluorescence was evaluated and values were normalized to the untreated control. The mean values are shown of three independent experiments ± standard deviation.

Gene expression profiling displayed increased levels of DDB1, DDB2, XPC, GADD45A and RRM2B, indicating the activation of the DNA damage response and repair system by the platinum drugs. We therefore investigated the repair of platinum-induced DNA damage on a functional level. Cells were treated with the respective drug for 2 h and ICP-MS was used to measure platinum bound to DNA directly after drug exposure and after a recovery period of 24 h. The amount of overall DNA platination was reduced to less than 40% within 24 h in case of damage induced by cisplatin and carboplatin, and to less than 30% for oxaliplatin-induced damage, indicating the ability for repair of DNA platinum lesions (Table 2). DNA damage induced by cisplatin, carboplatin and oxaliplatin consists to over 90% of intrastrand crosslinks between GG or GA [16]. However, a small percentage of interstrand crosslinks (ICLs) is also formed in response to treatment with the platinum drugs. As phosphorylation of H2AX can be used as a marker for the generation and processing of DNA ICLs [17,18], we measured overall fluorescence staining for γ-H2AX formation upon treatment with the respective platinum drug. Overall, γ-H2AX fluorescence measurement was applied, since cisplatin-induced foci are small and difficult to count accurately. γ-H2AX fluorescence were observed 24 h post-treatment with cisplatin and carboplatin and increased over time, while no fluorescence signal beyond background was detected following treatment of A498 cells with oxaliplatin (Figure 6A,B). 

### 2.6. Cell Cycle Regulation and Apoptosis 

**Figure 7:**
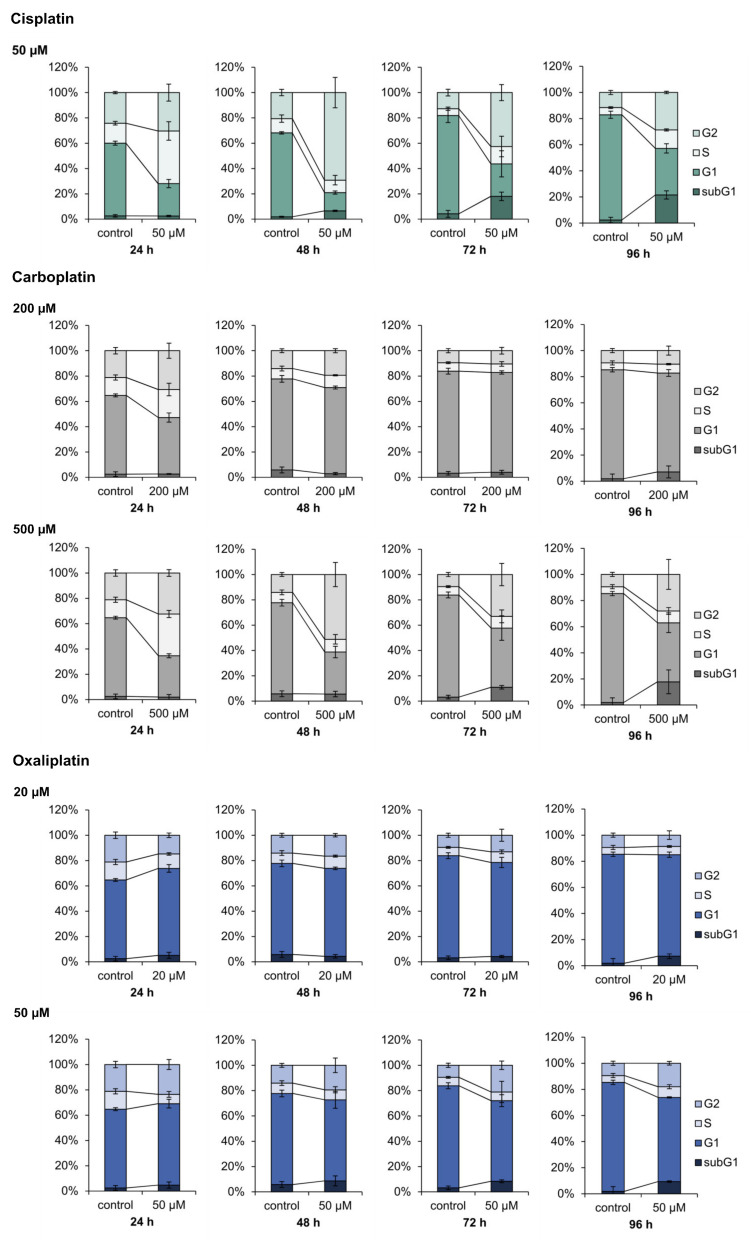
Analysis of the cell cycle distribution of A498 cell after treatment with cisplatin, carboplatin or oxaliplatin. Cells were incubated for 1 h with cisplatin (50 µM), carboplatin (200, 500 µM) or oxaliplatin (20, 50 µM) followed by postcultivation of 24, 48, 72 or 96 h. Cell cycle distribution was analyzed by DAPI (4′,6′-diamidino-2-phenylindole) staining via flow cytometry. The mean values are shown of three independent experiments of the percentage distribution of the cell cycle phases ± standard deviation. The data for cisplatin are taken from [8].

Induction of the cell cycle related genes CDKN1A, CCND1, PLK3 and PPMID and the apoptosis related genes BBC3, APAF1, PMAIP1 and TNFRSF10B suggested modulation of the respective pathway by the platinum drugs. DAPI staining and flow cytometry was therefore used for functional analysis of cell cycle regulation and apoptosis upon treatment with carboplatin and oxaliplatin, and the results were compared to data established for cisplatin [13]. A498 cells were treated with the respective compound for 1 h and cell cycle distribution was measured after a 24, 48, 72 and 96 h recovery period. Two different concentrations were chosen for carboplatin (200, 500 µM) and oxaliplatin (20, 50 µM). Cisplatin we used solely at a concentration of 50 µM, as cell cycle distribution upon cisplatin treatment has been described previously numerous times [19,20]. When used in equitoxic concentrations, carboplatin affected cell cycle distribution of A498 cells comparably to cisplatin (Figure 7). Treatment of the cells with carboplatin (500 µM) caused a S phase arrest after 24 h postcultivation, which was followed by a G_2_/M arrest after 48 h. The percentage of cells in the three different phases of the cell cycle then further declined until 96 h, while the percentage of cells in the subG_1_ phase increased. Cell cycle distribution was essentially the same after incubation of the cells with 50 µM cisplatin. Additionally, 200 µM carboplatin resulted in only a slight increase of the S phase as well as the G_2_/M phase after 24 h postcultivation. After 72 h postcultivation, no differences in the cell cycle distribution of the untreated control and the treated cells were observed. This might be explained by the low DNA platination levels observed upon carboplatin treatment due to its slower reaction kinetics compared to cisplatin, in addition to lower platinum accumulation (Table 2). In contrast to cisplatin and carboplatin, oxaliplatin did not show any substantial effect on cell cycle control and regulation. Incubation with 20 µM oxaliplatin caused a slight increase in the G_0_/G_1_ phase at the expense of cells in the G_2_/M phase. However, no further changes between the untreated control and the treated cells were observed up to 96 h post-treatment. Incubation with 50 µM oxaliplatin led to a slight decrease of the S phase after 24 h, while the percentages of cells in the other phases were slightly increased. The small increase of cells in the G_2_/M phase persisted until 96 h.

**Figure 8:**
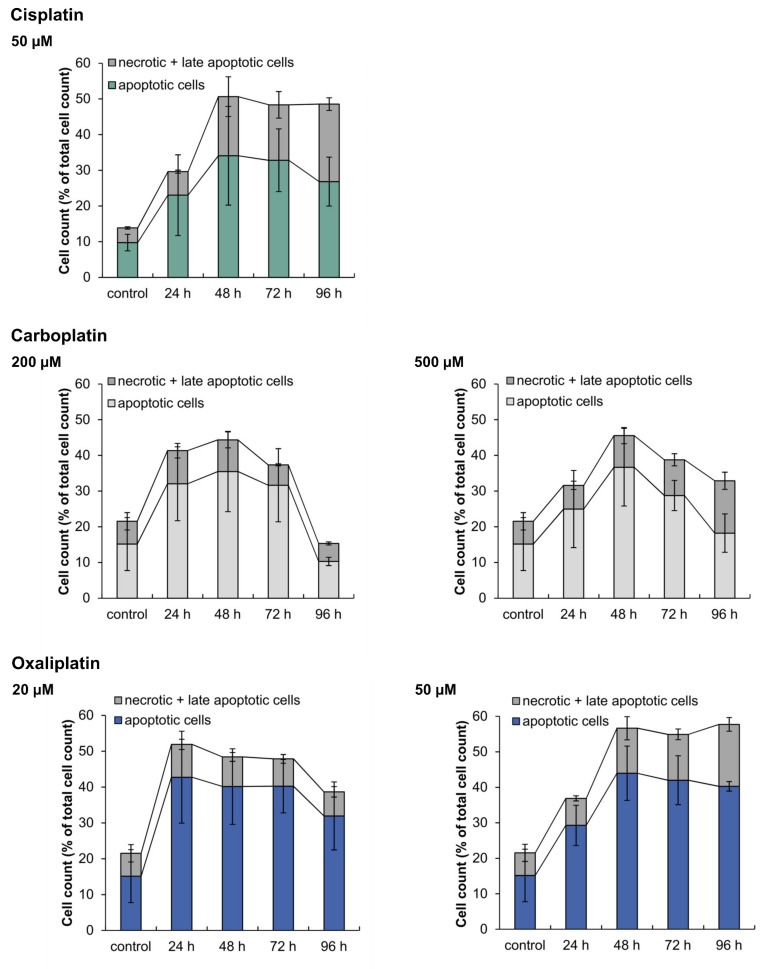
Analysis of apoptotic and necrotic cells after incubation of A498 cells with cisplatin, carboplatin or oxaliplatin. Cells were incubated for 1 h with cisplatin (50 µM), carboplatin (200, 500 µM) or oxaliplatin (20, 50 µM) followed by postcultivation of 24, 48, 72 or 96 h. To distinguish between apoptotic and necrotic + late apoptotic cells, cells were stained with Annexin V-FITC and propidium iodide (PI). Cell cycle distribution was then analyzed by flow cytometry. The mean values are shown of three independent experiments ± standard deviation. The data for cisplatin are taken from [8].

To analyze the induction of apoptosis on a functional level and to distinguish between apoptotic and necrotic cell death, cells were stained with annexin V-FITC and PI and fluorescence was measured by flow cytometry. A498 cells were incubated with carboplatin or oxaliplatin for 1 h followed by postcultivation for 24, 48, 72 and 96 h. The results were compared to cisplatin (Figure 8). All three platinum compounds caused the induction of apoptosis as the preferred mechanism of cell death. Following treatment with 50 µM cisplatin, the percentage of apoptotic cells increased after just 24 h postcultivation and continued up to 48 h, while the percentage of necrotic and late-apoptotic cells also increased. The level of dead cells in general remained the same at 72 h and 96 h, but the percentage of necrotic and late-apoptotic cells increased, whereas the percentage of apoptotic cells declined. The treatment of A498 cells with 200 µM carboplatin also led to an increase of apoptotic cells after 24 h postcultivation. However, after 96 h, the percentage of dead cells drastically declined, while the percentage of vital cells increased from 50 % to 80 %. Under these conditions, the cells probably repaired DNA damage and recovered. Nevertheless, the percentage of dead cells in general also declined after incubation with 500 µM carboplatin, but not as drastically as seen with 200 µM. Oxaliplatin also caused an increase in the percentage of apoptotic cells after 24 h postcultivation at both concentrations. After 96 h postcultivation, the treatment with 20 µM oxaliplatin led to a slight decline in the percentage of dead cells. This was not seen with an incubation of 50 µM oxaliplatin. Compared to carboplatin and oxaliplatin, cisplatin caused the highest percentage of necrotic cells.

## 3. Discussion

The platinum drugs cisplatin, carboplatin and oxaliplatin are widely used for cancer treatment, but the molecular mechanisms that mediate their anticancer activity are not fully elucidated. In particular, limited information is available regarding the mode of action of oxaliplatin. In the present study, the mechanistic activity of carboplatin and oxaliplatin was investigated in the A498 kidney cancer cell line and compared to observations for cisplatin [13]. The main outcome of our investigation was the finding that cisplatin and carboplatin share the same mechanism of activity, but oxaliplatin differs in its mode of action. The three platinum compounds were cytotoxic to A498 cells, with a stronger toxicity observed for cisplatin and oxaliplatin compared to carboplatin at equimolar concentrations. The lower toxicity of carboplatin compared to cisplatin has also been reported in previous studies in various cancer cells lines. It has been noted that carboplatin is less reactive than cisplatin, as it shows slower reaction kinetics regarding the aquation step. The chelate-leaving group of carboplatin is considerably more stable than the chloride groups of cisplatin, resulting in slower aquation and, hence, lower reactivity, yielding an approximately 10-fold slower rate of DNA adduct formation [21,22,23]. ICP-MS measurements confirmed lower levels of DNA platination in A498 cells upon treatment with carboplatin versus cisplatin. Oxaliplatin treatment also resulted in fewer DNA platination lesions compared to cisplatin, but exhibited a similar cytotoxic activity. This might be explained by observations that oxaliplatin-induced lesions are generally more cytotoxic than cisplatin lesions and show a greater inhibition of DNA synthesis [24]. Furthermore, DNA platination was also associated with intracellular platinum accumulation, with considerably lower platinum accumulation measured upon carboplatin treatment. Platinum-based compounds enter the cells via passive diffusion and by active transport via membrane transporters. Copper transporter 1 (Ctr1) and organic cation transporter 2 (OCT2) have been implicated in the active uptake of platinum-based compounds. The differences in intracellular accumulation might therefore be explained with the different affinities of the platinum compounds to these membrane transporters. Regarding Ctr1, cisplatin was found to have a higher affinity towards the transporter than carboplatin, while oxaliplatin appears to be taken up by Ctr1 only at low concentrations, whereas uptake at higher concentrations was independent of Ctr1 [25,26]. With regard to OCT2, various studies suggested a role of OCT2 in the uptake of cisplatin and oxaliplatin, but not of carboplatin [27,28]. OCT2 is primarily expressed in the kidney, which might explain low intracellular platinum accumulation following carboplatin treatment of A498 kidney cancer cells and higher levels of accumulation upon treatment with cisplatin or oxaliplatin. However, intracellular accumulation of platinum not only depends on uptake transporters, but also on efflux transporters. Two important efflux transporters which are especially expressed in the kidney are MATE1 and MATE2-K. It could be shown that oxaliplatin represented a substrate for both transporters, whereas cisplatin was not transported by MATE2-K and at reduced levels by MATE1 [28,29]. Therefore, varied affinities of the platinum drugs to the efflux transporters might explain the observed differences in the intracellular accumulation of cisplatin and oxaliplatin.

Systematic gene expression profiling showed a dose-dependent impact of the platinum compounds on genes related to genomic stability. The repair-related genes *GADD45A*, *XPC*, *DDB1*, *DDB2* and *RRM2B* were upregulated in response to the compounds; the strongest effect was observed for oxaliplatin. The modulation of genes related to DNA repair suggests activation of the cellular DNA repair machinery. ICP-MS was applied to monitor DNA repair at the functional level. Considering whole DNA platination, ICP-MS measurements revealed reduced amounts of platinum bound to DNA after a repair period of 24 h, confirming repair of the overall DNA platination damage induced by the respective platinum compound. Platinum compounds induce various kinds of DNA lesions, particularly intrastrand crosslinks between two adjacent guanine or two guanine-adenine bases which make up more than 90% of the induced lesions [30,31,32]. Cisplatin-induced intrastrand crosslinks are mainly removed by NER, the main DNA repair pathway dealing with bulky helix-distorting lesions [33,34]. As the same intrastrand crosslinks are formed by carboplatin, NER consequently also participates in the repair of carboplatin-induced DNA damage. It was also shown that cisplatin- and oxaliplatin-induced DNA lesions are repaired with similar kinetics by the NER system [35]. Indicative of the activation of NER, transcript levels of *XPC*, *DDB1*, *DDB2* and *GADD45A*, which directly or indirectly take part in NER [36,37], were upregulated by cisplatin, carboplatin and oxaliplatin in the present study. In addition to intrastrand crosslinks, a small percentage of interstrand crosslinks (ICLs) is induced by the platinum drugs [30,38]. To monitor the repair of platinum-induced ICLs, γ-H2AX formation was used as a marker associated with ICL repair [17]. We observed staining for γH2AX in response to cisplatin and carboplatin, but not oxaliplatin in A498 cells. In agreement with our observations, no staining for γ-H2AX was observed in lymphoma cells or colon cancer cells after treatment with oxaliplatin, whereas cisplatin treatment resulted in γ-H2AX foci [39,40]. On the other hand, γ-H2AX foci were detected in oxaliplatin-treated Chinese hamster ovary cells; the level, however, was small compared to that of cisplatin-treated cells [41]. As γ-H2AX formation serves as a marker associated with the induction and processing of ICLs [17], the lack of formation of γ-H2AX upon oxaliplatin treatment might suggest that either oxaliplatin does not induce ICLs or oxaliplatin-induced ICLs are not repaired. However, an induction of ICLs by oxaliplatin was reported by Woynarowski and colleagues who studied oxaliplatin-induced DNA damage in naked and cellular DNA. They observed that even though oxaliplatin, in general, induced less DNA platination damage compared to cisplatin, with mainly DNA intrastrand crosslinks being formed, few DNA interstrand crosslinks were nonetheless observed [42]. One might speculate that oxaliplatin-induced ICLS are less efficiently repaired by the cellular machinery. Structural differences between ICLs induced by cisplatin versus oxaliplatin have been reported [38]. The bulky DACH ligand of oxaliplatin sticks out of the helix and leads to a different bending of the DNA; as a result, oxaliplatin-induced ICLs might be poorly recognized or even prevented from binding by some DNA repair enzymes [32,43]. ICLs are removed by ICL repair, a process which is less well understood than NER [44]. In mammalian cells, NER and homologous recombination take part in ICL repair, although additional pathways involving DNA polymerases may also contribute to this [45]. Biochemical studies implicate the repair proteins BRCA1, BRCA2, RAD51 in homologous recombination [46]. Decreased transcript levels of *BRCA1*, *BRCA2* and *RAD51*, as observed by gene expression profiling, might be an indication of a lack of processing of oxaliplatin-induced ICLs. Interestingly, despite differences in the amount of DNA platination, as shown in the present study and by Woynarowski and colleagues [42], oxaliplatin and cisplatin exhibit similar cytotoxicity. One can speculate that oxaliplatin exerts its cytotoxic activity not only through DNA platination, but also additional mechanisms. Bruno and colleagues suggest that ribosome biogenesis stress might be the key player in the mode of action of oxaliplatin [39]. Ribosome biogenesis stress can lead to a profound activation of p53 which, in turn, induces cell death [47]. This might also explain why the protein level of p53 was more increased after treatment with oxaliplatin than cisplatin or carboplatin (Figure 5). Elevated p53 protein levels upon drug treatment also suggest activation of p53, which was confirmed by increased transcript levels of p53 target genes *MDM2*, *XPC*, *DDB2*, *GADD45A*, *RRM2B*, *CDKN1A*, *PLK3*, *PPM1D*, *APAF1*, *BBC3*, *PMAIP1* and *TNFRSF10B* [15]. The involvement of p53 in the mode of action of platinum drugs has been reported (Köberle, unpublished observations) [48,49]. However, it has also been reported that platinum drugs can induce cell death in the absence of functioning p53 [13,50]. In our study, p53 appeared to play a role in the mode of action of all three platinum-based compounds, as shown by increased expression of p53 target genes and p53 protein stabilization. A498 cells harbor wild type p53, which might suggest that in cell lines with functioning p53, platinum-based drugs will use a p53-dependent DNA damage response.

As reactive oxygen species (ROS) and inflammation are often the cause of unwanted side effects of chemotherapeutic drugs [51], the impact of cisplatin, carboplatin and oxaliplatin on inflammatory and oxidative stress response has been investigated. Gene expression analysis revealed that the three platinum drugs had only little impact on genes associated with oxidative stress response. Studies on the functional level regarding the induction of ROS or oxidative DNA damage also did not show any unambiguous results. The lack of an oxidative stress response, therefore, does not implicate ROS as major determinants of platinum-induced side effects For the clinically approved platinum drugs, severe side effects are reported. The dose-limiting side effect of cisplatin is nephrotoxicity, while carboplatin induces severe myelosuppression, and neurotoxicity is caused by oxaliplatin. Inflammation mediated by chemokines such as IL-8 is thought to play a key role in cisplatin-induced nephrotoxicity [52,53,54]. An inflammatory response in oxaliplatin-induced neuropathies is still questionable [55], but IL-8 seems to play a role in the mode of action of oxaliplatin [56]. The inflammation marker *IL-8*, which was dose-dependently upregulated by the platinum compounds, is transcriptionally controlled by NF-κB [57]. During inflammatory processes a correlation between the expression of *NFκB*, *IL-8* and *VEGFA*, which belongs to the vascular endothelial growth factor family (VEGF), was observed [58]. In our gene expression analysis, both cisplatin and oxaliplatin caused a concentration-dependent increase in the NF-κB gene *NFKB2* and in *VEGFA*, while carboplatin did not show any modulation of *NFKB2* and *VEGFA*. One might therefore speculate that cisplatin as well as oxaliplatin cause an inflammatory response in A498 cells, while carboplatin does not. However, further studies need to substantiate this.

With respect to genes associated with cell cycle regulation, increased transcript levels of *CDKN1A*, *PLK3* and *PPM1D*, all of which indicative of cell cycle arrest [59,60,61], were observed in response to cisplatin, carboplatin or oxaliplatin. In addition, oxaliplatin also upregulated *SIRT2*, which also serves as indication of cell cycle arrest [62]. On the other hand, oxaliplatin also enhanced the expression of *CCND1* and *EGFR*, indicating cell cycle progression rather than cell cycle arrest [63]. Most strikingly, oxaliplatin led to a pronounced repression of *E2F1*. E2F1 serves as a transcription factor which is involved in cell cycle regulation. It appears to be essential for the entry into S phase [64]. Kiyonari and colleagues also detected a downregulation of E2F1 after the treatment of colon cancer cell lines HCT116 and LoVo with oxaliplatin [48]. As the downregulation of E2F1 was associated with a decreased expression of enzymes involved in thymidylate synthase, they suggest that these observations might explain the synergistic effect of oxaliplatin and 5-fluorouracil (5-FU) in the treatment of advanced colon carcinoma [48]. In accordance with the upregulation of *CDKN1A*, *PLK3* and *PPM1D*, cell cycle arrest was observed upon treatment with cisplatin and carboplatin at the functional level, which is in agreement with published data [65]. Oxaliplatin, on the other hand, showed no substantial impact on cell cycle distribution in A498 cells kidney cancer cells. For colon cancer cells, only a weak effect of oxaliplatin on the cell cycle was reported [20]. Furthermore, a possible effect of oxaliplatin on cell cycle distribution might be cell type specific [66]. Nevertheless, in our studies using a kidney cancer cell line, we did not observe a substantial impact by oxaliplatin on the cell cycle, whereas cisplatin and carboplatin induced significant change in the cell cycle distribution.

Treatment of A498 cells with the three platinum complexes modulated genes related to apoptosis. Induction of the pro-apoptotic genes *APAF1*, *BBC3*, *PMAIP1* and *TNFRSF10B* was observed upon treatment with cisplatin and oxaliplatin, indicating apoptotic signaling via the intrinsic and extrinsic cascade. Regarding carboplatin, *APAF1*, *BBC3* and *PMAIP1* were upregulated, while the transcript level of *TNFRSF10B* was not affected, suggesting induction of apoptosis via the intrinsic cascade cy carboplatin. Accordingly, for the platinum compounds, induction of apoptosis was confirmed by flow cytometry on the functional level, in agreement with previous investigations in various cancer cells [67,68,69]. Flow cytometry also revealed induction of necrosis, especially in the case of cisplatin, but apoptosis appeared to be the primary mechanism of cell death.

## 4. Materials and Methods 

### 4.1. Figures, Platinum-Based Chemotherapeutics

Cisplatin solution was provided by the Municipal Clinic Karlsruhe with a concentration of 1 g/L. Carboplatin and oxaliplatin solutions were purchased from Accord Healthcare Limited (Middlesex, Great Britain) at concentrations of 10 mg/mL and 5 mg/mL, respectively.

### 4.2. Cell Culture

The studies were performed using the renal cell carcinoma cell line A498 [70] which was purchased from Deutsche Sammlung von Mikroorganismen und Zellkulturen (DSMZ). A498 cells were cultivated as monolayers in RPMI-1640 medium supplemented with 10% fetal calf serum, penicillin (100 U/mL) and streptomycin (100 µg/mL) (Sigma Aldrich, Steinheim, Germany) at 37 °C in a humidified atmosphere of 5% CO_2._

### 4.3. Cytotoxicity Studies

A relative cell count was performed by seeding 2.5 × 10^5^ cells in duplicate in 6 cm dishes. After cultivation for 24 h, cells were treated for 1 h with cisplatin (10, 20, 40 and 80 µM), carboplatin (40, 80, 160, 320 and 500 µM) or oxaliplatin (20, 40, 80 and 160 µM) followed by postincubation for 72 h. Subsequently, cell count was undertaken using a CASY^®^ cell counter (CASY^®^ TTC Cell Counter & Analyzer System). Relative cell count was performed by normalizing the cell count of surviving cells from the treated samples to the untreated control expressed as percentage. For the determination of colony formation ability, 600 cells were seeded in triplicates in 6 cm dishes. After cultivation for 24 h, cells were incubated with carboplatin (50, 100, 150 and 200 µM) or oxaliplatin (5, 10, 15 and 20 µM) for 1 h and cultivated for 11–12 days in fresh medium. Colonies were fixed for 5 min with ice cold 96% ethanol, stained with 5% Giemsa (Roth, Karlsruhe Germany) and those consisting of 50 or more cells were counted. Colony formation in treated dishes was expressed as a percentage of colony formation in the untreated controls. RCC and CFA were performed in three independent experiments. CFA data for cisplatin (5, 10, 15 and 20 µM) were previously described in [13].

### 4.4. Intracellular Platinum Accumulation

Intracellular platinum accumulation was analyzed via atomic absorption spectroscopy (AAS). First, 2 × 10^6^ cells were seeded in 10 cm dishes und cultivated for 24 h. In contrast to previous assays, cells were subsequently treated for 2 h with 50 µM platinum-based compound. A 2-h incubation time was chosen due to the limits of platinum detection observed for AAS. The total cell count was measured and cells were pelleted. The cell pellets were digested with a solution of 30% H_2_O_2_ and 65% HNO_3_ (1:1 (v:v)) (Roth, Karlsruhe, Germany) and evaporated. After digestion, the residue was dissolved in 0.2% HNO_3_ and used to measure the platinum amount with a PinAAcle 900 T (Perkin Elmer, Waltham, MA, USA). Platinum was determined at a wavelength of 265.94 nm in a graphite furnace. The furnace temperature program consisted of two drying stages of 120 °C for 30 s and 140 °C for 45 s, a pyrolysis stage of 1300 °C for 20 s and an atomization step at 2400 °C for 5 s, as well as a heating step of 2500 °C for 5 s. Intracellular platinum accumulation was calculated as Pt ng/10^6^ cells, and was performed in three independent analyses. The data of platinum accumulation measured after cisplatin treatment were taken from [13].

### 4.5. DNA Platination

Due to the limits of detection of platinum by AAS, DNA platination was analyzed by inductively coupled plasma mass spectroscopy (ICP-MS). First, 3 × 10^6^ cells were seeded in 20 cm dishes and cultivated for 24 h, followed by incubation with 50 µM platinum-based chemotherapeutic agent for 2 h. Cells were then harvested either immediately via centrifugation or after a 24 h postincubation period. Each sample was seeded in duplicate but was combined after DNA extraction to ensure the presence of enough DNA material for measurements above the determination limit of the ICP-MS, particularly for measurement of carboplatin and oxaliplatin DNA platination. DNA was extracted by suspending the pellets in 100 µL warm (37 °C) TE-Buffer containing 10 mM Tris-HCl (pH 8.0) and 6.25 mM EDTA (Roth, Karlsruhe, Germany). This solution was mixed with 900 µL warm (37 °C) extraction buffer (pH 8.0) consisting of 0.01 M Tris, 0.1 M EDTA, (Roth, Karlsruhe, Germany) 0.5% SDS (AppliChem, Darmstadt, Germany) and 20 µg/mL heat inactivated RNase A (Roth, Karlsruhe, Germany). The samples were incubated under agitation at 37 °C for 1 h 15 min, followed by supplementation with proteinase K (20 µg/mL) (Merck Millipore, Darmstadt, Germany) and incubation for another 3 h under agitation at 50 °C. After phenol/chloroform/isoamyl alcohol (25:24:1) extraction (2×) and chloroform/isoamyl alcohol (24:1) extraction, DNA was precipitated with 1/20 volume sodium acetate (10 M) and 2 volume ethanol (absolute), washed four times with ethanol (70%), dried and dissolved in 500 µL bidestilled water for quantification. DNA quantification was performed in duplicate with a NanoQuant plate at a Tecan Infinite M200 Pro in UV range and checked for purity. Afterwards, samples were evaporated and digested with nitric H_2_O_2_. The residue was dissolved in 2 mL 0.2% HNO_3_ and used for analysis with ICP-MS. Platinum amount was analyzed with a Thermo Scientific XSERIES 2 with CCT (collision cell technology) and a plasma performance of 1400 W. The most frequently isotopes of platinum (^194^Pt and ^195^Pt) were analyzed and the mean of both determinations was calculated. The amount of DNA platination was calculated in nmol Pt/g DNA and determined in three independent experiments.

### 4.6. Gene Expression Profiling by High-Throughput RT-qPCR

For gene expression profiling, 1 × 10^6^ cells were seeded in 10 cm dishes and cultivated for 24 h. An untreated control was included and samples were seeded in duplicate. After cultivation, cells were treated for 1 h carboplatin (50, 100, 200, 300, 400 and 500 µM) or oxaliplatin (20, 50, 100, and 150 µM) followed by 24 h postcultivation and cell harvesting via centrifugation. To generate gene expression profiles, a high-throughput RT-qPCR was performed after RNA isolation using a Fluidigm dynamic array on a BioMark™ system. Experiments and evaluation were performed according to [14]. The gene expression profiles of cisplatin treatment (10, 20 and 50 µM) were taken from [13].

### 4.7. Immunblotting of p53 

To determine the p53 protein levels, 1 × 10^6^ cells were seeded in 10 cm dishes and cultivated for 24 h. Cells were incubated for 1 h with carboplatin (50, 100, 200, 300, 400, 500 µM) or oxaliplatin (10, 20, 50, 100, 150 µM). After treatment, cells were cultivated for an additional 24 h and harvested via centrifugation. Protein extraction was carried out by lysing the cell pellets in 120 µL lysis buffer containing 50 mM Tris-HCl (pH 7.5), 250 mM NaCl, 1 mM EDTA, 0.1% Triton X-100 (Roth, Karlsruhe, Germany) and 1× protease inhibitor (cOmplete Mini, Roche, Mannheim, Germany) for 30 min on ice under agitation, followed by centrifugation for 20 min at 4 °C and 14,000 rpm. The supernatant was recovered and the protein concentration was analyzed by the Bradford method (Bio-Rad Protein Assay, Munich, Germany).

For SDS-PAGE, 50 µg protein extract of each sample was used and separated in 10% gel. Protein transfer was carried out overnight at 4 °C on an Amersham Hybond P PVDF membrane (GE Healthcare, Buckinghamshire, UK) in Tris-glycine buffer (25 mM Tris-HCl, 192 mM glycine, 20% methanol). Unspecific protein binding sites were blocked by incubating the membrane for 1 h in PBST (Tween 0.005%) with 5% milk powder. Membranes were incubated with 1/2000 diluted monoclonal p53 antibody (clone DO-7, DAKO, CA, USA) in 5% milk powder-PBST-solution overnight at 4 °C. Afterwards, membranes were washed with PBST and incubated with 1/2000 diluted antimouse IgG-HRP antibody (Santa Cruz Biotechnology, Santa Cruz, CA, USA) in 5% milk powder-PBST-solution overnight at 4 °C. As a loading control ERK2 was chosen. The antibodies used were polyclonal 1/2000 diluted ERK2 (C-14, Santa Cruz Biotechnology, Santa Cruz, CA, USA) and 1/2000 antirabbit IgG-HRP (Santa Cruz Biotechnology, Santa Cruz, CA, USA). Membranes were treated as previously stated. Protein visualization was performed by ECL-detection according to the manufacturers protocol using a LAS-3000 Imaging System (Fuji, Minato, Japan). Semiquantitative evaluation was carried out with the program Aida Image Analyzer v.3.27. The detection of p53 protein was performed in three independent experiments. The results for cisplatin (10, 20, 50 µM) were previously described by [13].

### 4.8. Analysis of Apoptosis and Cell Cycle Distribution via Flow Cytometry 

First, 2 × 10^5^ A498 cells were seeded in duplicate in 6-cm dishes and cultivated for 24 h. Cells were then treated for 1 h with 200 and 500 µM carboplatin or 20 and 50 µM oxaliplatin, followed by postcultivation for 24, 48, 72 and 96 h. Besides an untreated control, 24 h incubation with 500 nm staurosporine was used as a positive control to check for functionality of the method. Untreated and positive controls were included for each time point. After postcultivation, the media and cells were transferred into a 15 mL tube and centrifuged at 4 °C and 1300 rpm for 4 min. The supernatant was discarded and the cells were resuspended in 2 mL PBS from which 1 mL was used for the determination of apoptosis and 1 mL for analysis of cell cycle distribution.

For analysis of the cell cycle distribution, cells were fixed with 3 mL ice cold ethanol, which was added slowly under constant mixing to avoid clumping. For complete fixation, cells were stored at −20 °C overnight. The fixed cells were then centrifuged at 4 °C and 4000 rpm for 5 min, the supernatant was discarded and cells were washed with 1 mL PBS, followed by centrifugation. Cells were resuspended in 300 µL DAPI (4′,6′-diamidino-2-phenylindole) staining solution (Partec, Münster, Germany) and kept on ice for 30 min in the dark. Fluorescence was measured with a BD LRSII Fortessa flow cytometer (BD, Heidelberg, Germany) with a violet laser of 488 nm excitation and a bandpass filter of 450/50 nm. To determine the cell cycle distribution, counts were plotted over the fluorescence signal in a histogram.

For the determination of apoptosis, 1 mL cell suspension was centrifuged, the supernatant discarded and the cells were resuspended in a mix of 200 µL Ringer solution (147 nM NaCl, 402 nM KCl, 297 nM CaCl_2_) + 0.25 µL propidium iodide (PI) (62.5 ng/mL) + 1 µL annexin V-FITC (450 ng/mL) and kept on ice for 30 min in the dark. While PI was used to determine necrotic/late apoptotic cells, annexin V-FITC was used to stain apoptotic cells. Fluorescence was again analyzed by flow cytometry using the same laser as stated above and a bandpass filter of 695/40 nm for the PI signal and of 530/30 nm for the FITC signal. Since the absorption spectra of PI and FITC overlapped, compensation was required prior to analysis. To distinguish between vital, apoptotic and nectrotic and late apoptotic cells, the PI signal was plotted in a dot plot over the FITC signal. The determinations of apoptosis and cell cycle distribution were carried out in three independent experiments. The results for treatment with 50 µM cisplatin were taken from [13].

### 4.9. Staining for γ-H2AX 

A coverslip was placed in a 3.5 cm Petri dish, and 1 × 10^5^ cells were seeded into the dish and cultivated for 24 h. Samples were prepared in duplicate. Following 24 h of cultivation, cells were incubated for 1 h with 20 and 50 µM cisplatin or oxaliplatin, respectively, or 200 and 500 µM carboplatin. After treatment, cells were postcultivated for 24, 48 or 72 h. At each time point, samples were washed with PBS and fixed with ice cold 99.9% methanol for 5 min. Methanol was discarded and cells were again washed with PBS three times for 5 min. Following fixation, cells were permeabilized with a solution of 0.2% Triton X-100 in PBS for 10 min on a shaker and afterwards washed again with PBS three times for 5 min. Cells were blocked for 30 min in 1% BSA in PBST solution and then incubated with 1/1000 diluted mouse monoclonal antiphospho-Histone H2AX (Ser139, clone JBW301, Merck, Darmstadt, Germany). The coverslips facing the cell side were placed on a droplet containing the antibody in a wet chamber and incubated for 1 h. After three washing steps with PBS, cells were incubated with the secondary antibody. A polyclonal antimouse antibody coupled with Alexa Fluor^®^ 488 from Invitrogen (Darmstadt, Germany) diluted 1/1000 in blocking solution was used as a secondary antibody. Cells were incubated as stated previously for 1 h in the dark and washed again three times. The coverslips were then placed on a slide with Vectashield HardSet Antifade Mounting Medium with DAPI (Vector Laboratories, Burlingame, CA, USA) and sealed. Since platinum-induced foci are small and difficult to count accurately, overall γ-H2AX fluorescence was analyzed by fluorescence microscopy with a Axio Imager Z2 (Carl Zeiss, Jena, Germany) and the software ZEN. Overall γ-H2AX fluorescence was evaluated with the software Axio Vision 4.8 and values were normalized to the untreated control. Studies were performed in three independent experiments.

## 5. Conclusions

Our investigation revealed that carboplatin and cisplatin share the same mode of action. Carboplatin induces identical DNA lesions to cisplatin [23], which lead to a comparable DNA damage response, as revealed by gene expression profiling and functional analysis in the present study. The lower toxicity of carboplatin, which was observed in A498 kidney cancer cells and various other cells including ovarian cancer cells [4], can be explained by lower levels of DNA accumulation and DNA platination. Lower DNA accumulation is most likely linked to different affinities to the membrane transporter Ctr1 observed for carboplatin and cisplatin [25]. In addition, a lower aquation rate due to more stable ligands also contributed to the reduced level of DNA platination upon carboplatin treatment [21,22,23]. The mechanism of action of oxaliplatin, however, differed significantly from that of cisplatin and carboplatin, particularly with respect to induction and processing of DNA damage. Oxaliplatin exhibited similar cytotoxicity to cisplatin, despite lower levels of DNA platination which were associated with lower cellular platinum accumulation, possibly due to platinum export via the membrane transporters MATE1 and MATE2-K [28,29]. It is reported that oxaliplatin-induced lesions are more cytotoxic than cisplatin lesions and show a greater inhibition of DNA synthesis [24]. Furthermore, oxaliplatin-induced ICLs appear to be less efficiently processed by the cellular machinery, as we observed using staining for γ-H2AX, which serves as a marker associated with induction and processing of ICLs [17]. This might be explained by structural differences of ICLs induced by cisplatin versus oxaliplatin [38]. On the transcriptional level, repression of specific DNA repair genes, especially related to repair pathways dealing with ICLs, was observed solely for oxaliplatin. Taken together, a difference in platinum lesions, particularly ICLs, and processing of the ICLs most likely results in a differing mechanism of action of oxaliplatin compared to cisplatin and carboplatin. This might be of interest with regard to cisplatin tumor cell resistance. As carboplatin shows cross resistance to cisplatin due to the same mode of action, oxaliplatin might still be active in tumors which are unresponsive to cisplatin and carboplatin. Future studies will determine whether the range of platinum-treated tumors might be expanded by the application of oxaliplatin.

## Figures and Tables

**Table 1:** IC_50_ values of cisplatin, carboplatin and oxaliplatin determined in A498 cells by relative cell count (RCC) and colony formation ability (CFA).

Platinum-Based Compound	RCC [µM]	CFA [µM]
cisplatin	27 ± 4.1	6 ± 3.4
carboplatin	273 ± 6.9	153 ± 6.1
oxaliplatin	36 ± 5.8	12 ± 2.0

**Table 2:** Platinum accumulation and DNA platination in A498 cells following incubation for 2 h with 50 µM of the platinum-based compound. The mean values are shown from three independent determinations ± standard deviation (* two independent determinations). The result of cisplatin accumulation is taken from [8].

Platinum-Based Compound	Accumulation (ng Pt/10^6^ cells)	DNA Platination (nmol Pt/g DNA)	
		0 h	24 h
cisplatin	23 ± 4.6	383 ± 252.2	149.5 ± 76.6
carboplatin	4.8 ± 0.2	17.0 ± 12.7 *	6.5 ± 0.7 *
oxaliplatin	14.9 ± 1.0	149.5 ± 76.6	36 ± 5.5

## Supplementary Materials

Supplementary Materials can be found at https://www.mdpi.com/1422-0067/21/18/6928/s1.

## Abbreviations

AAS	atomic absorption spectroscopy
CFA	colony formation ability
DDR	DNA damage response
DSB	double strand break
ICL	interstrand crosslink
ICP-MS	inductively coupled plasma mass spectroscopy
MMR	mismatch repair
NER	nucleotide excision repair
RCC	relative cell count
SSB	single strand break
