# Supplementary material for: Comparative Study of the Mode of Action of Clinically Approved Platinum-Based Chemotherapeutics
Source: Int J Mol Sci. 2020 Sep 21;21(18):6928. doi: 10.3390/ijms21186928 (PMC7555145; doi:10.3390/ijms21186928)
Supplement: Supplementary file 1 [file ijms-21-06928-s001.pdf]

International Journal of *Molecular Sciences*

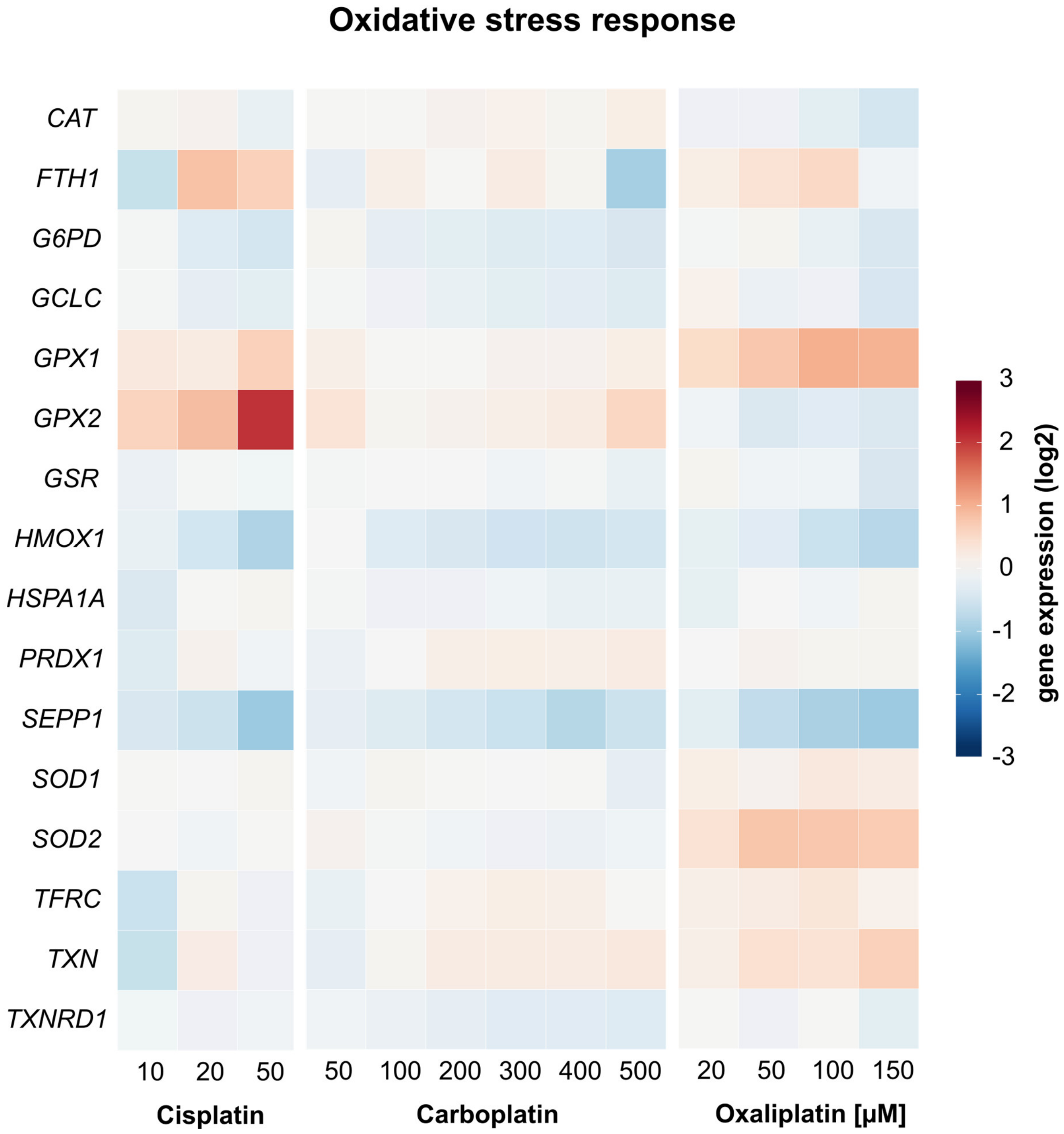

Supplementary figure 1: Gene expression analysis of (oxidative) stress related genes after treatment of A498 cells with cisplatin, carboplatin or oxaliplatin. Cells were treated for 1 h with cisplatin (10, 20, 50 µM), carboplatin (50, 100, 200, 300, 400, 500 µM) or oxaliplatin (20, 50, 100, 150 µM). This was followed by a post cultivation of 24 h. Afterwards gene expression was determined via high-throughput RT-qPCR. Shown are the log2 mean values of three independent experiments normalized to the untreated control whereby the control equals 0. The data for cisplatin are taken from [8].
